# ESM1 promotes angiogenesis in colorectal cancer by activating PI3K/Akt/mTOR pathway, thus accelerating tumor progression

**DOI:** 10.18632/aging.204559

**Published:** 2023-03-07

**Authors:** Liqun Yang, Zhigang Dong, Shuyu Li, Tieliang Chen

**Affiliations:** 1 General Surgery, Tangshan Fengnan District Hospital, Fengnan, Tangshan 063300, China; 2 Two Divisions of The Cardiovascular Duct, Affiliated Hospital of North China University of Science and Technology, Lubei, Tangshan 063300, China; 3 General Surgery, Tangshan Union Hospital, Lunan, Tangshan 063300, China

**Keywords:** ESM1, PI3K/Akt/mTOR pathway, CRC, angiogenesis

## Abstract

Background: This study aimed to explore the influence of endothelial cell-specific molecule 1 (ESM1) expression on colorectal cancer (CRC) cells and preliminarily analyze its possible mechanism, so as to lay a foundation for research about potential biological targets of CRC.

Methods: First, CRC cells were transfected with ESM1-negative control (NC), ESM1-mimic and ESM1-inhibitor and randomly assigned to ESM1-NC group, ESM1-mimic group and ESM1-inhibitor group, respectively. Then the cells were harvested at 48 h after transfection for subsequent experiments.

Results: The results manifested that after up-regulation of ESM1, the distance of CRC SW480 and SW620 cell lines migrating to the scratch center rose notably, and the number of migrating cells, basement membrane-penetrating cells, colonies formed and angiogenesis was increased overtly, indicating that ESM1 overexpression can promote tumor angiogenesis in CRC and accelerate tumor progression. Combined with results of bioinformatics analysis, the molecular mechanism by which ESM1 promoted tumor angiogenesis in CRC and accelerated tumor progression was explored through suppressing the protein expression of phosphatidylinositol 3-kinase (PI3K). Western blotting revealed that after intervention with PI3K inhibitor, the protein expressions of phosphorylated PI3K (p-PI3K), phosphorylated protein kinase B (p-Akt) and phosphorylated mammalian target of rapamycin (p-mTOR) were decreased evidently, and the protein expressions of matrix metalloproteinase-2 (MMP-2), MMP-3, MMP-9, Cyclin D1, Cyclin A2, VEGF, COX-2 and HIF-1α subsequently declined.

Conclusion: ESM1 may promote angiogenesis in CRC by activating the PI3K/Akt/mTOR pathway, thus accelerating tumor progression.

## INTRODUCTION

Colorectal cancer (CRC) ranks third among the most frequently occurring malignancies worldwide, and its incidence rate shows an upward trend. According to statistics, there are approximately 1 million new cases and 0.55 million deaths every year globally [1]. Although prominent advances have been made in the application of molecular targeted drugs combined with chemotherapy in recent years, the prognosis of patients with metastatic CRC remains unsatisfactory [2, 3]. Besides, the mechanism of CRC metastasis is still elusive. Therefore, searching for molecular markers closely associated with metastasis to monitor the metastatic process of CRC is urgently needed, and then appropriate measures can be taken in time. Angiogenesis is a physiological process by which new blood vessels are generated from pre-existing blood vessels [4]. The balance between pro-angiogenic signals and anti-angiogenic signals has generally been considered to modulate angiogenesis [5]. If this balance is disrupted, it will greatly impact tumor development [6, 7]. Hence, angiogenesis is crucial in promoting tumor progression and is co-regulated by a series of angiogenesis stimulators and inhibitors in tumor cells and their stroma [3, 8]. Vascular endothelial growth factor (VEGF), also known as vascular permeability factor (VPF), is a highly specific pro-vascular endothelial cell growth factor, which has the effects of increasing vascular permeability and promoting extracellular matrix degeneration and vascular endothelial cell migration, proliferation and angiogenesis. Cyclooxygenase-2 (COX-2) is able to promote angiogenesis by inducing VEGF and basic fibroblast growth factor expressions. Hypoxia-inducible factor-1α (HIF-1α) can induce VEGF expression in hypoxia, and inhibition of HIF-1α can reduce angiogenesis.

Endothelial cell-specific molecule 1 (ESM1) located on chromosome 5q11.2 is proven to be a crucial regulator of endothelial cell function [9, 10]. In normal tissues, ESM1 is expressed in vascular endothelial cells, distal renal tubular epithelial cells, bronchi and pulmonary submucosal glands. Recent studies have revealed that ESM1 may participate in the occurrence and development of such cancers as head and neck squamous cell carcinoma (HNSCC), hepatocellular carcinoma (HCC) and bladder cancer (BLCA) in human [11, 12]. ESM1 overexpression is closely associated with drug resistance to chemotherapy in breast cancer (BRCA) [13] and has certain impact on the clinical outcome of advanced gastric cancer after chemotherapy [14, 15], indicating that ESM1 is of potential clinical value in cancer patients.

However, there are few studies about the role of ESM1 in CRC. In particular, the mechanism of the effect of ESM1 on CRC cells has not yet been entirely elucidated. Therefore, combined with bioinformatics analysis, the effects of ESM1 expression on angiogenesis in CRC and biological behaviors of CRC cells were investigated and its possible mechanism of action was preliminarily analyzed in this study, so as to lay a foundation for research about potential biological targets of CRC.

## MATERIALS AND METHODS

### Cell culture and grouping

CRC SW480 and SW620 cell lines and human normal colorectal cells (CCD-841CoN, IM-H477) were purchased from Nanjing KeyGen Biotech Co., Ltd. SW480 and SW620 cells were inoculated into L15 medium containing 10% fetal bovine serum (FBS) and 1% penicillin-streptomycin and incubated in an incubator at 37°C with 5% CO_2_. The cells in logarithmic growth phase were inoculated into a 6-well plate and the medium was changed normally prior to transfection to ensure that the cells were in good condition. Upon reaching approximately 70% confluence, CRC cells were transfected with ESM1-negative control (NC), ESM1-mimic and phosphatidylinositol 3-kinase (PI3K)-inhibitor, respectively, according to the instructions of Lipofectamine 2000 kit (Invitrogen, USA), which were then randomly assigned to ESM1-NC group, ESM1-mimic group, ESM1-NC + PI3K inhibitor group and ESM1-mimic + PI3K inhibitor group. Herein, LY294002 (5 μL), which is a commonly-used PI3K inhibitor and can permeate cells and specifically inhibit PI3K, was utilized. Then the cells were harvested at 48 h after transfection for subsequent experiments.

### Wound healing assay

First, 2 × 10^5^ cells from each group were inoculated into a 24-well plate, followed by conventional incubation in a constant-temperature incubator at 37°C with 5% CO_2_. Upon reaching 100% confluence, a wound was made in the middle of the culture plate by drawing a line via the tip of a 10 μL pipette, and the old medium was replaced by serum-free medium. The scratch was photographed and recorded under an inverted microscope at 0, 24 and 48 h, and the width of scratch was measured by ImageJ software [16].

### Detection of CRC cell migration and invasion abilities via Transwell assay

In cell migration assay, the cell suspension (200 μL) of SW480 and SW620 cell lines (2 × 10^5^/mL) after transfection and Dulbecco’s modified Eagle medium (DMEM, 600 μL) were added into the Transwell upper chamber and lower chamber, respectively. After 24 h of culture, the cells were fixed in paraformaldehyde and stained with crystal violet solution, followed by counting for the number of migrating cells. With regard to invasion assay, the diluted matrigel was plated in the Transwell upper chamber for 5 h, and subsequent procedures were carried out in the same way as those in migration assay to measure the number of invading cells [17].

### Colony formation assay

First, 2× DMEM and upper-layer and lower-layer agarose gels were prepared. The lower-layer gel was mixed with an equivalent volume of 2× DMEM, and then quickly added into a 6-well plate, followed by standing at room temperature until solidification. The upper-layer gel was mixed with an equivalent volume of 2× DMEM, and the cultured cells in each group were added into the upper-layer gel with 1 × 10^4^ cells in each well. After the upper-layer gel was solidified, the cells were incubated in an incubator for 2–3 weeks and then stained with crystal violet solution. Subsequently, they were washed with phosphate-buffered saline (PBS), photographed and counted, and the colony formation ability of cells in each group was determined [18].

### Methyl thiazolyl tetrazolium (MTT) assay

A single cell suspension (2 × 10^4^ cells per mL) was prepared with culture medium containing 10% FBS and seeded into a 96-well plate with a volume of 200 μL per well. Under the same general culture conditions, the cells were cultured for 3 days. Then MTT solution (5 mg/mL with PBS) was added to each well for 20 μL. After 4 h, the incubation was discontinued, and the supernatant was carefully aspirated. The suspension cells were centrifuged and the supernatant was discarded. Next, 150 μL of DMSO was added per well and then shaken for 10 min to fully melt the crystals. The absorbance was measured at a wavelength of 490 nm using a microplate reader. The results were recorded, and the cell growth curve was drawn with the time as the abscissa and the absorbance value as the ordinate [19].

### Measurement of cell cycle via flow cytometry

The supernatant was discarded and the cells were washed 2 times with pre-cooled PBS. Then they were mixed well with a little PBS remaining in the precipitate, and 70% ethanol pre-cooled at −20°C was slowly added and shaken well to avoid cell adhesion. Next, the cells were placed in a −20°C refrigerator for more than 4 h and centrifuged at 50 g for 5 min, and the supernatant was discarded. The cells were washed twice with 2 mL of PBS and added with 500 μL of PBS (1 × 10^6^ cells) containing 50 μg/mL PI and 100 μg/mL RNaseA. The cells were incubated at 4°C protected from light for 15–30 min (PI could be directly formulated with PBS as a working concentration, and RNaseA was now added). Then the analysis was performed [20].

### Co-culture of cells and tube formation assay

Effector cells were seeded into the Transwell chamber and cultured for 24 h. Target cells were seeded into the cell culture plate and cultured for 24 h. After overnight incubation, the culture medium was removed from the Transwell chamber and plate, and the new culture medium was added to the lower chamber. The Transwell chamber was placed into the cell culture plate and the new culture medium was added for detection after 48 h of incubation. Then the Transwell chamber was removed and the culture medium was aspirated from the cell culture plate. The cell culture plate was washed twice with PBS and fixed in 4% paraformaldehyde.

Human umbilical vein endothelial cells (3B-11 cells) were cultured in endothelial growth medium-2 (EGM-2). After digestion with trypsin, the cells were inoculated into a 24-well plate coated with matrigel at 5 × 10^4^ cells per well. At 48 h after culture, the cells were digested with 1 mL of trypsin-EDTA. After digestion was complete, the serum-containing culture medium was added to terminate digestion. The supernatant was taken to incubate 3B-11 cells in an incubator at 37°C for 3–5 h. Then the supernatant was discarded and the cells were gently washed once with PBS. Next, 250 μL of culture solution was added in each well. Images were captured within 1 h and analyzed by ImageJ software, and the number of angiogenesis was calculated [21].

### Subcutaneous nodules in nude mice

The SW480 cells in the logarithmic growth phase were collected. The luciferase gene marker was transfected with ESM1-NC and ESM1-mimic, respectively. Under standard conditions, the cells were centrifuged, and approximately 5 × 10^6^ cells obtained were dissolved in 0.1 mL of DMEM culture medium for later use. Mice (SPF-grade athymic Balb/c-nu/nu nude mice, male, 4–5 weeks old, body weight: 15–20 g) were anesthetized with 3% pentobarbital sodium and then observed.

### Immunofluorescence double staining

Tissue frozen sections 95% alcohol fixed at room temperature for 10 min, PBS washed 3 times for 5 min each, 1% Titon-10 treated at room temperature for 10 min PBS washed 3 times, 10% goat serum blocked at 37°C for 1 h, goat serum removed, anti-CA199 antibody (Mouse monoclonal to CA199, ab270734), incubated overnight at 4°C, room temperature for 30 min, PBS washed 3 times (steps protected from light), FITC-labeled goat anti-mouse secondary antibody (Goat Anti-Mouse IgG H&L, ab6785) incubated at 37°C for 1.5 hours, PBS washed 3 times, 10% goat serum sealed again at 37°C for 1 h, anti-CD31 antibody (Rabbit polyclonal to CD31, ab28364) incubated at 37°C for 1 h, PBS washed 3 times, donkey Anti-Rabbit IgG H&L (Alexa Fluor^®^ 647) (ab150075) Incubate at 37°C for 1 hour, seal the tablets with the extinction solution, and take pictures under fluorescence microscopy.

### Q-PCR

TRIzol test to extract total RNA from brain tissues, after the BeyoRT^™^II cDNA First Strand Synthesis Kit synthesizes cDNA, perform qPCR experiments with SYBR Green. The qPCR reaction conditions were predenaturation at 95°C for 5 min, 95°C for 30 s, 65°C for 45 s, and 40 cycles ring, 72°C for 5 min. The forward primer for ESM1 is 5′-GGGAGAAACTTGCTACCGCA-3′ and the reverse primer is 5′-GCCATGTCATGCTCTTTGCAG-3′.

### Western blotting

SW480 and SW620 cells were lysed in RIPA lysate on ice for 30 min. The BCA method was utilized to detect the concentration of total protein in cells and tissues. After protein concentrations were adjusted to be basically consistent, proteins were separated by SDS-PAGE, transferred onto a membrane, and blocked with 5% skim milk for 2 h, followed by incubation with primary antibodies against phosphorylated PI3K (p-PI3K) (ab278545, Abcam), total PI3K (t-PI3K) (ab40776, Abcam), phosphorylated protein kinase B (p-Akt) (ab8805, Abcam), total Akt (t-Akt) (ab38449, Abcam), phosphorylated mammalian target of rapamycin (p-mTOR) (ab109268, Abcam), total mTOR (t-mTOR) (ab134903, Abcam), matrix metalloproteinase-2 (MMP-2) (ab92536, Abcam), MMP3 (ab92519, Abcam), MMP-9 (ab76003, Abcam), Cyclin D1 (ab16663, Abcam), Cyclin A2 (ab181591, Abcam), VEGF (ab32152, Abcam), COX-2 (ab179800, Abcam), HIF-1α (ab179483, Abcam), ESM1 (ab103590, Abcam) and glyceraldehyde-3-phosphate dehydrogenase (GAPDH) (ab181602, Abcam) on a shaker at 4°C overnight. Then the membrane was washed and incubated with secondary antibodies (1:5000) on the shaker at room temperature for 2 h, followed by color development with ECL reagent. Target protein bands were photographed by a gel imager, and the experiment was repeated three times. Finally, the relative expression of proteins was calculated by ImageJ software through comparison with the internal reference.

### Statistical analysis

SPSS 22.0 software was adopted for statistical analysis, and GraphPad Prism 5.0 was used for processing. Normally distributed measurement data were expressed by mean ± standard deviation $\text{(}\;\bar{\chi }\pm \text{s)}\text{.}$ Independent samples *t*-test was employed for comparison between two groups, and one-way analysis of variance for comparison among groups. *p* < 0.05 or *p* < 0.01 represented statistically significant differences.

## RESULTS

### The high expression of ESM1 in sw480 and sw620 cells demonstrates successful ESM1-mimic transfection

Through Q-PCR detection, we found that the relative expression of ESM1 in the ESM1-mimic group was significantly higher than in the ESM1-NC group in sw480 and sw620 cells. Successful transfection of ESM1 in the ESM1-mimic group was described (Supplementary Figure 1A, 1B).

### ESM1 enhanced migration and invasion abilities of CRC cells

**Figure 1:**
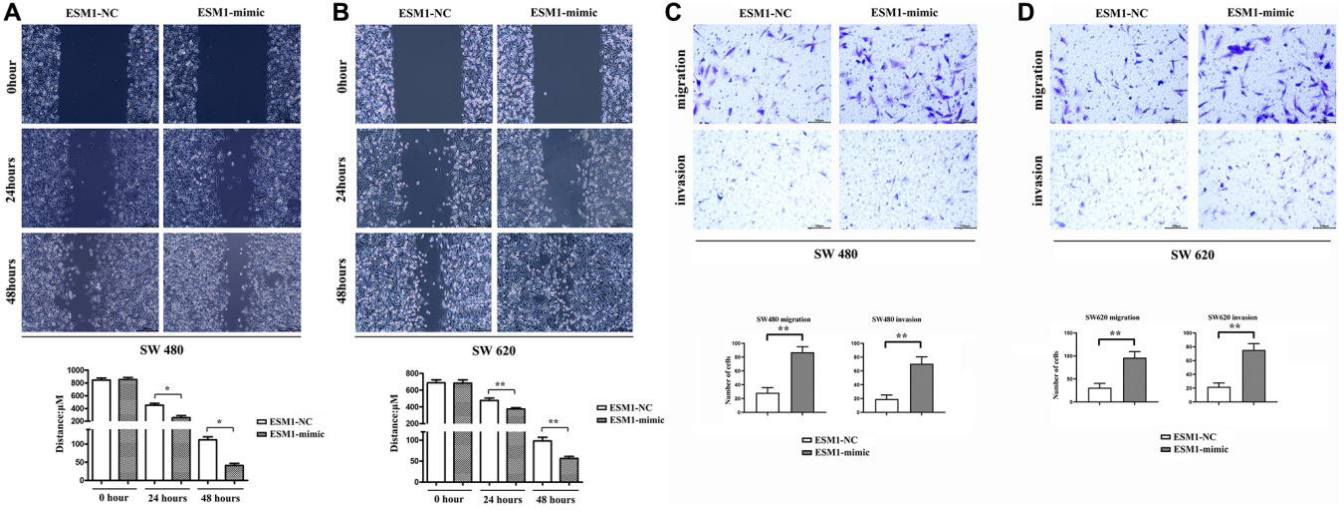
**Detection of migration and invasion abilities in SW480 and SW620 cells.** (**A**) Distances of migration at 0, 24 and 48 h after scratch formation in SW480 cells. (**B**) Distances of migration at 0, 24 and 48 h after scratch formation in SW620 cells. (**C**) Number of migrating and invading SW480 cells. (**D**) Number of migrating and invading SW620 cells. ^*^p < 0.05 and ^**^p < 0.01 vs. ESM1-NC group.

**Figure 2:**
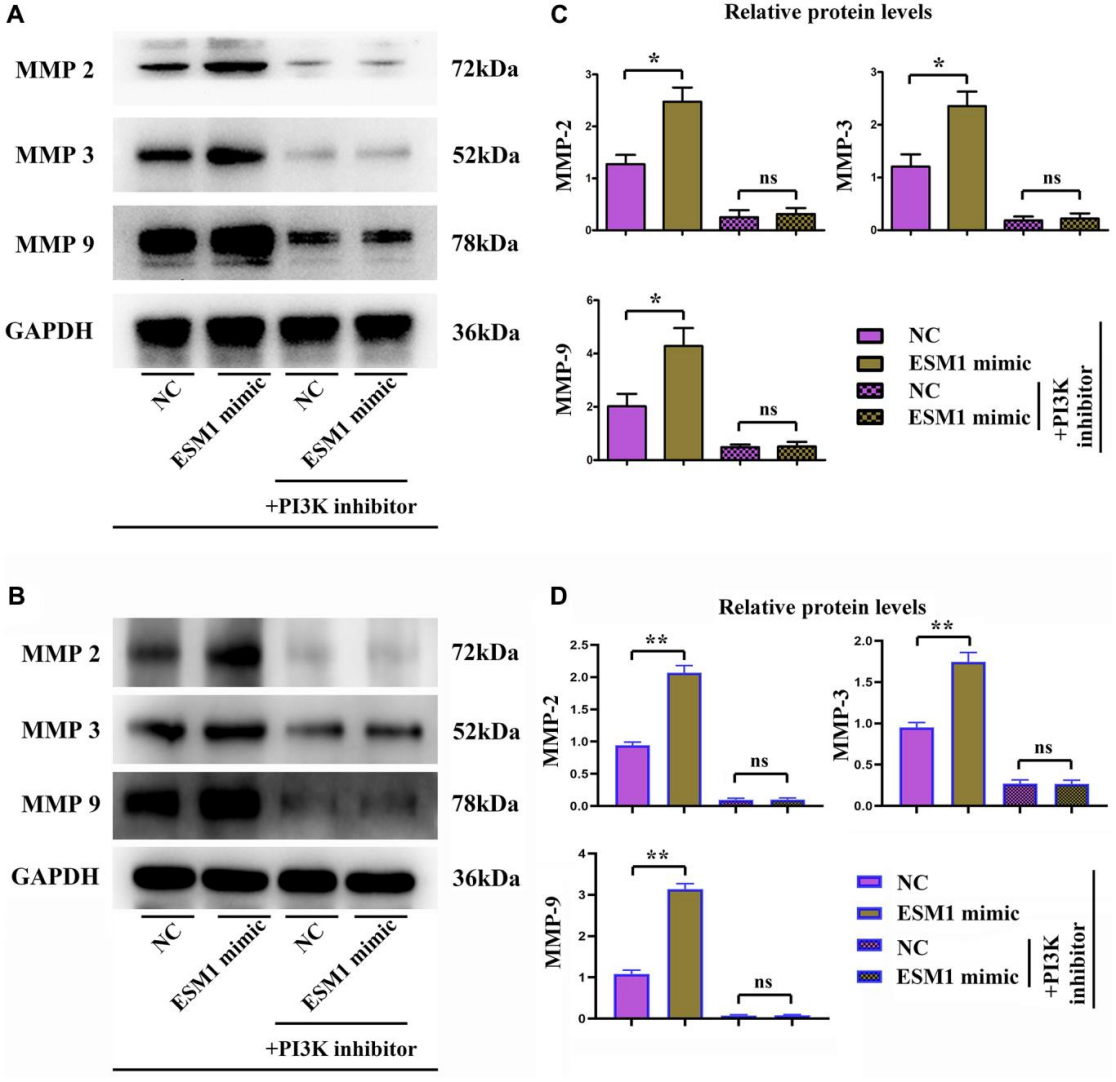
**Expressions of MMPs in each group.** (**A**, **B**) Relative protein expressions of MMP-2, MMP-3 and MMP-9 in ESM1-NC group, ESM1-mimic group, ESM1-NC + PI3K inhibitor group and ESM1-mimic + PI3K inhibitor group. (**C**, **D**) Statistics for MMP protein expressions. ^*^p < 0.05 and ^**^p < 0.01 vs. ESM1-NC group.

The results of wound healing assay manifested that ESM1-mimic group had a significantly smaller width of cell scratch than ESM1-NC group at 24 and 48 h, indicating that the distance of tumor cells migrating to the scratch center rose notably (Figure 1A, 1B). On the contrary, the width of cell scratch in ESM1-inhibitor was distinctly larger than that in ESM1-NC group. Similarly, the results of Transwell assay indicated that, compared with that in ESM1-NC group, the number of migrating SW480 and SW620 cells and basement membrane-penetrating cells was increased markedly in ESM1-mimic group (Figure 1C, 1D). Consistently, Western blotting revealed that in contrast to those in ESM1-NC group, the protein expressions of MMP-2, MMP-3 and MMP-9 were overtly elevated in ESM1-mimic group. The above findings suggest that ESM1 can enhance the migration and invasion abilities of CRC cells (Figure 2A–2D).

### ESM1 enhanced proliferation ability of CRC cells

**Figure 3:**
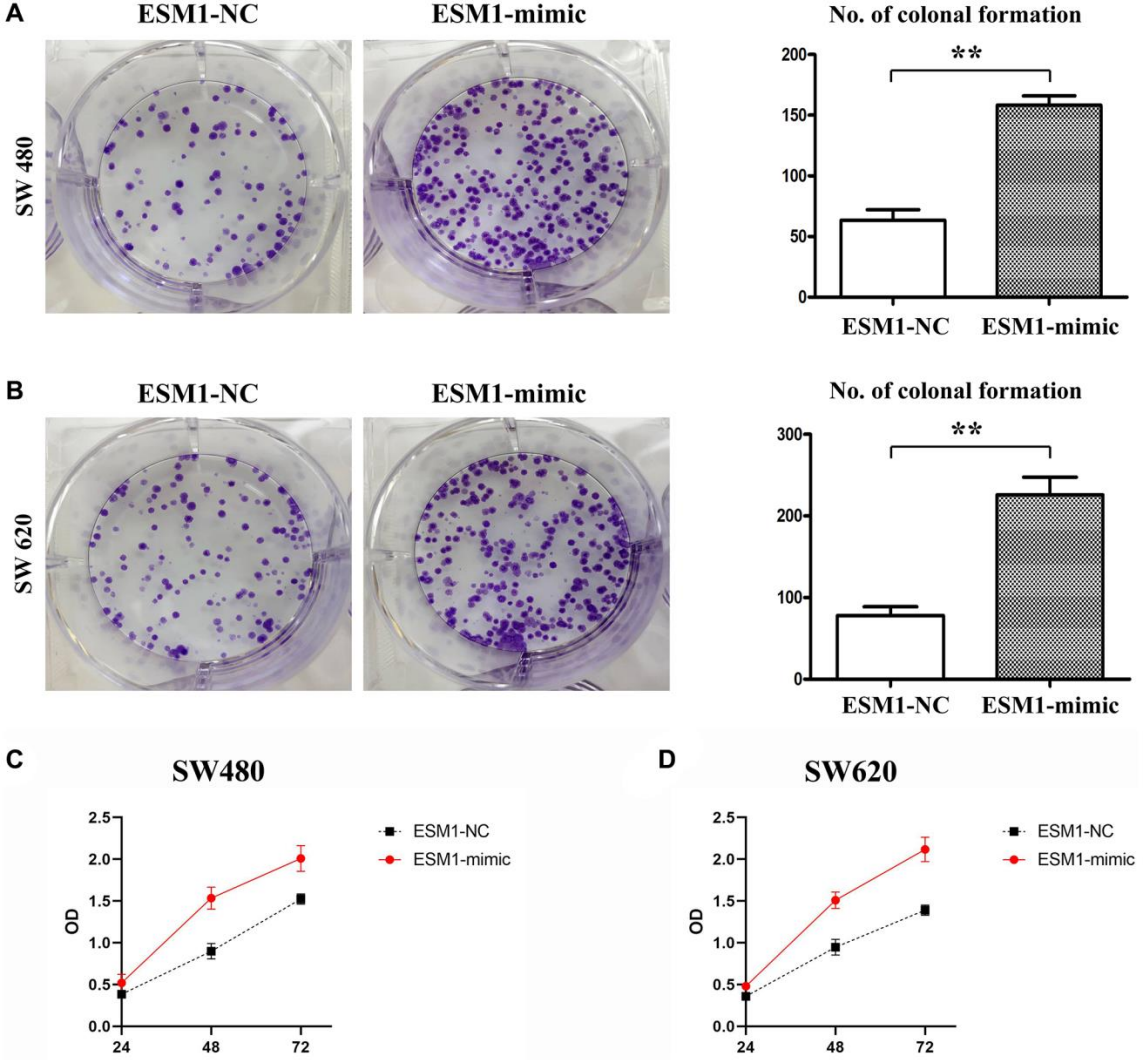
**Proliferation of SW480 and SW620 cells in each group.** (**A**) Number of colonies formed in SW480 cells transfected with ESM1-NC and ESM1-mimic. (**B**) Number of colonies formed in SW620 cells transfected with ESM1-NC and ESM1-mimic. (**C**) Detection of relative viability of SW480 cells via MTT assay. (**D**) Detection of relative viability of SW620 cells via MTT assay. ^*^p < 0.05 and ^**^p < 0.01 vs. ESM1-NC group.

**Figure 4:**
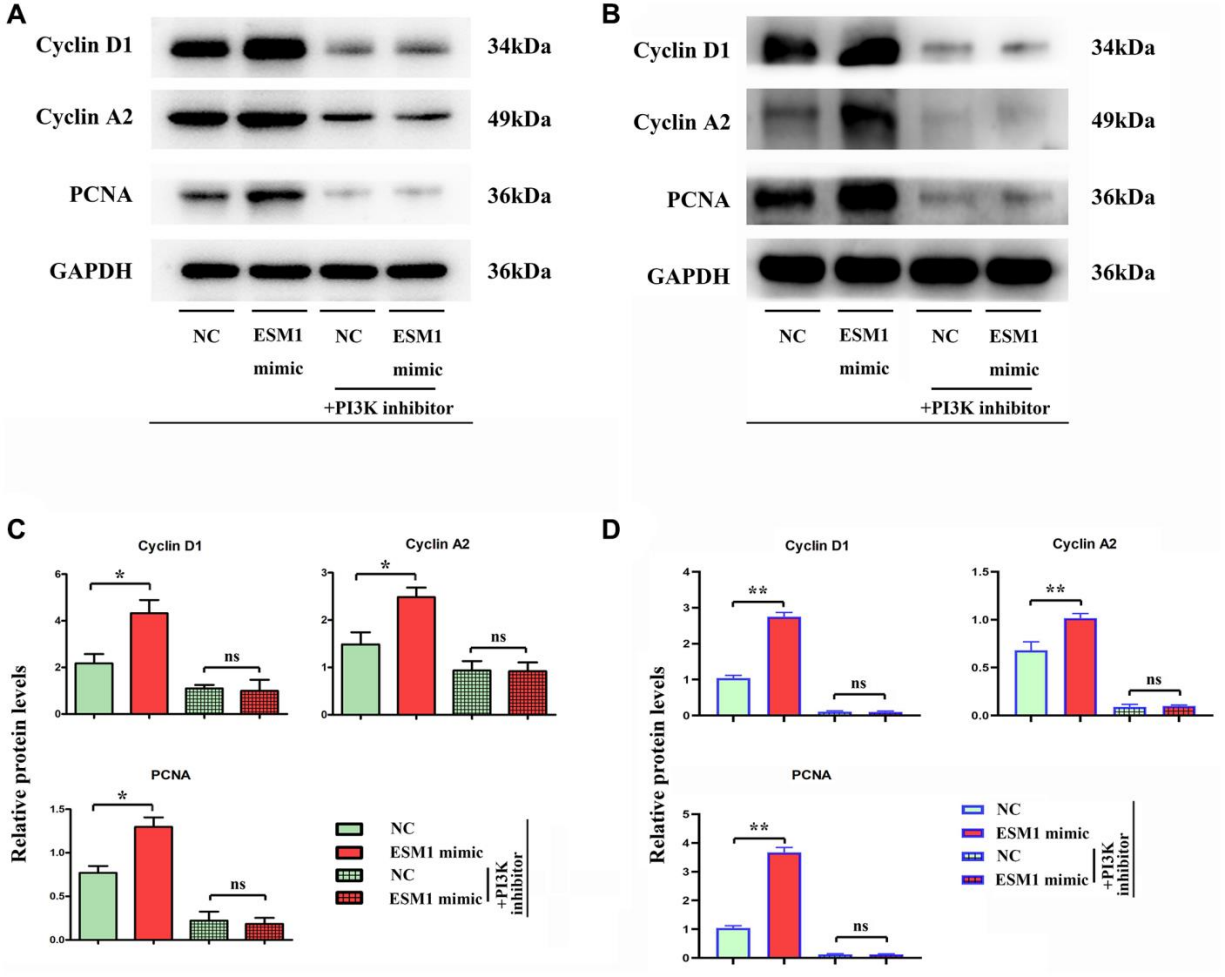
**Relative protein expressions of Cyclin D1 and Cyclin A2 in SW480 and SW620 cells.** (**A, B**) Relative protein expressions of Cyclin D1 and Cyclin A2 in ESM1-NC group, ESM1-mimic group, ESM1-NC + PI3K inhibitor group and ESM1-mimic + PI3K inhibitor group. (**C, D**) Statistics for Cyclin D1 and Cyclin A2 protein expressions. ^*^p < 0.05 and ^**^p < 0.01 vs. ESM1-NC group.

The results of colony formation assay showed that the number of colonies formed by SW480 and SW620 cell lines was raised after ESM1 overexpression *in vitro*, indicating that ESM1 can enhance the proliferation ability of CRC cells (Figure 3A–3D). It was found by MTT assay that the relative viabilities of SW480 and SW620 cells could be enhanced by ESM1-mimic. Furthermore, the alteration of proliferation-related protein expressions in SW480 cells was determined by Western blotting, and it could be seen that the protein expressions of Cyclin D1 and Cyclin A2 in SW480 and SW620 cell lines were remarkably higher in ESM1-mimic group than those in ESM1-NC group (Figure 4A–4D), indicating that ESM1 overexpression can enhance the proliferation ability of CRC cells.

### ESM1 promoted angiogenesis in CRC

**Figure 5:**
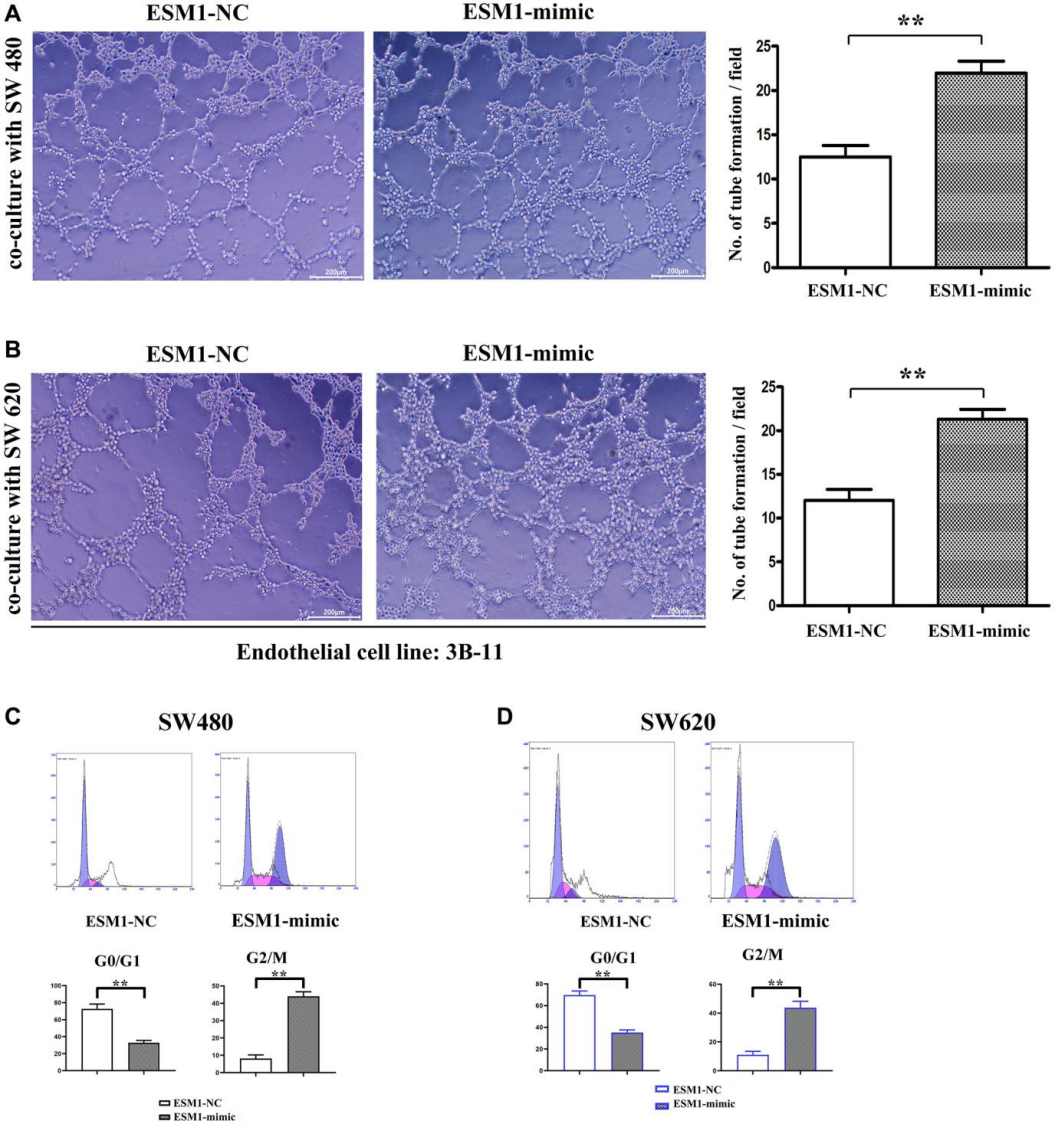
**Endothelial angiogenesis ability under SW480 and SW620 stimulation, and detection of effect of ESM1 on the proliferation of SW480 and SW620 cells via flow cytometry.** (**A**) Number of endothelial cell tube formation in 3B-11 cells co-cultured with SW480 cells transfected with ESM1-NC and ESM1-mimic. (**B**) Number of endothelial cell tube formation in 3B-11 cells co-cultured with SW620 cells transfected with ESM1-NC and ESM1-mimic. (**C**, **D**) Flow cytometry reveals that ESM1-mimic can significantly increase the ratio of G2 phase cells and promote cell proliferation. ^*^p < 0.05 and ^**^p < 0.01 vs. ESM1-NC group.

**Figure 6:**
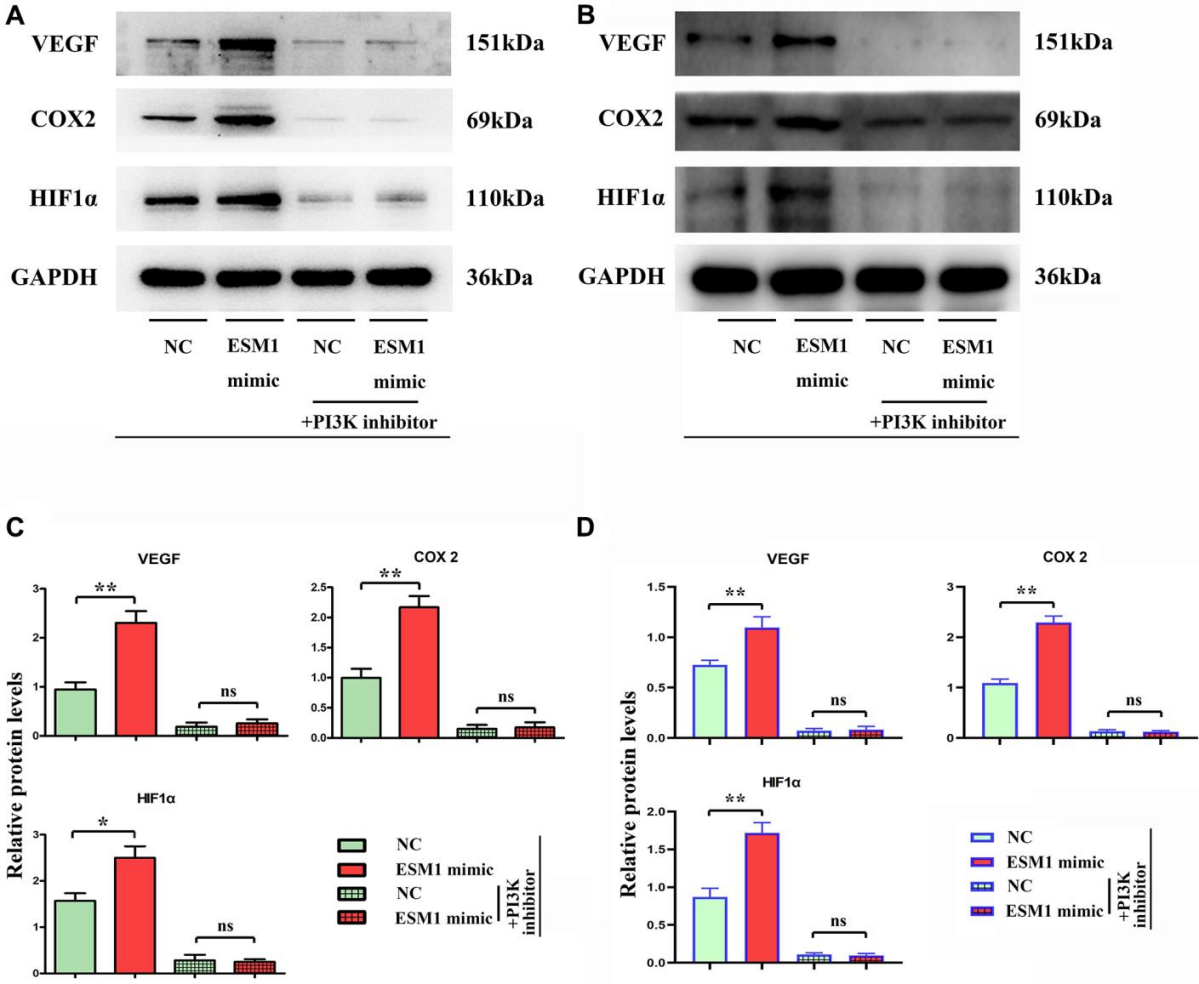
**Relative expressions of angiogenesis-associated proteins in each group.** (**A**, **B**) Relative protein expressions of VEGF, COX-2 and HIF-1α in ESM1-NC group, ESM1-mimic group, ESM1-NC + PI3K inhibitor group and ESM1-mimic + PI3K inhibitor group. (**C**, **D**) Statistics for VEGF, COX-2 and HIF-1α protein expressions. ^*^p < 0.05 and ^**^p < 0.01 vs. ESM1-NC group.

The results of tube formation assay denoted that compared with that in ESM1-NC group, the number of angiogenesis rose in ESM1-mimic group but notably declined in ESM1-mimic + PI3K inhibitor group, indicating that the supernatant of SW480 and SW620 cells with ESM1 overexpression can promote angiogenesis in 3B-11 cells (Figure 5A, 5B). Through flow cytometry, it was found that ESM1-mimic could increase the number of G2 phase cells. It is explained that ESM1 can promote the proliferation ability of SW480 and SW620 cells (Figure 5C, 5D). Consistently, the results of Western blotting implied that in comparison with those in ESM1-NC group, the protein expressions of VEGF, COX-2 and HIF-1α were prominently raised in SW480 and SW620 cells in ESM1-mimic group (Figure 6A–6D). These findings elucidate that ESM1 can promote angiogenesis in CRC.

### ESM1 contributed to angiogenesis in CRC by activating PI3K/Akt/mTOR pathway, thus accelerating tumor progression

**Figure 7:**
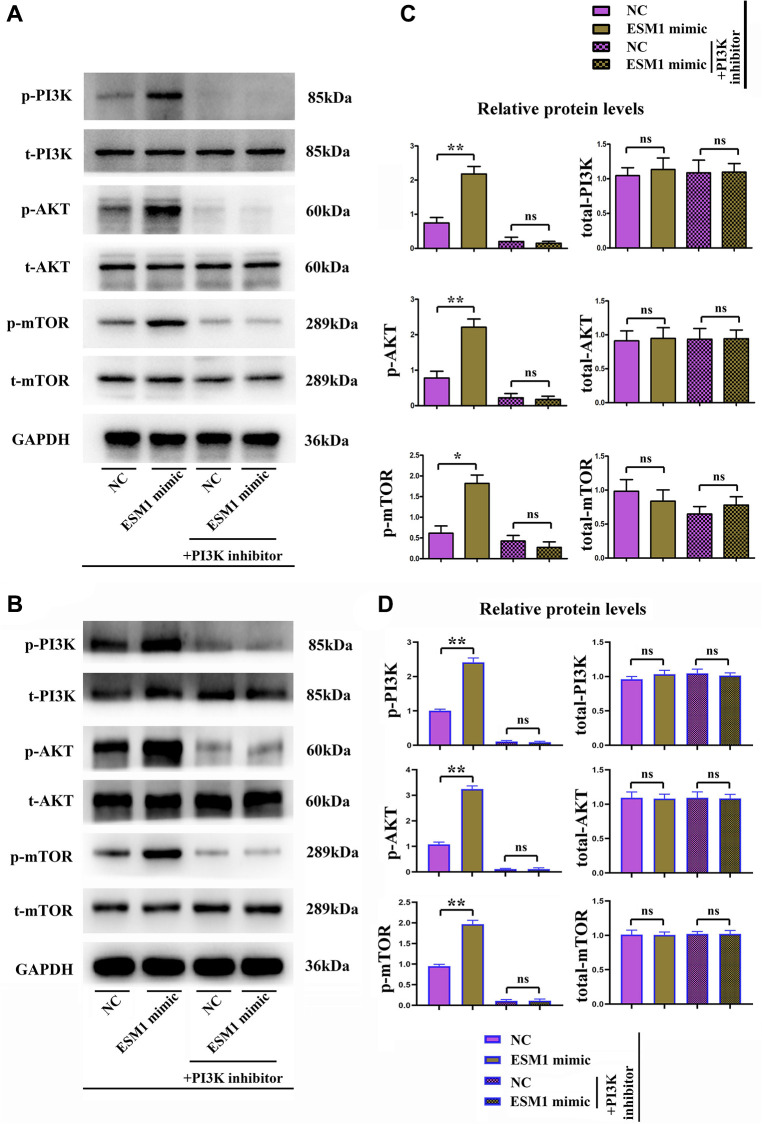
**Activated/phosphorylated protein levels of the PI3K/Akt/mTOR signaling pathway in each group.** (**A**, **B**) Phosphorylated and total protein levels of PI3K, Akt and mTOR in ESM1-NC group, ESM1-mimic group, ESM1-NC + PI3K inhibitor group and ESM1-mimic + PI3K inhibitor group. (**C**, **D**) Statistics for phosphorylated and total protein levels of PI3K, Akt and mTOR. ^*^p < 0.05 and ^**^p < 0.01 vs. ESM1-NC group.

Biological behavior experiments on SW480 and SW620 cells demonstrated that ESM1 can promote angiogenesis in CRC and accelerate the migration, invasion and proliferation of CRC cells. Western blotting demonstrated that the protein expressions of p-PI3K, p-Akt and p-mTOR in SW480 cells in ESM1-mimic group were significantly higher than those in ESM1-NC group. Then after intervention with PI3K inhibitor, the mechanism of action was further investigated. It was found that the protein expressions of p-PI3K, p-Akt and p-mTOR were decreased evidently, and the protein expressions of MMP-2, MMP-3, MMP-9, Cyclin D1, Cyclin A2, VEGF, COX-2 and HIF-1α subsequently declined (Figure 7A–7D). The above-mentioned findings indicate that ESM1 may promote angiogenesis in CRC by activating the PI3K/Akt/mTOR pathway, thus accelerating tumor progression.

### ESM1 promoted the growth and proliferation of CRC cells

**Figure 8:**
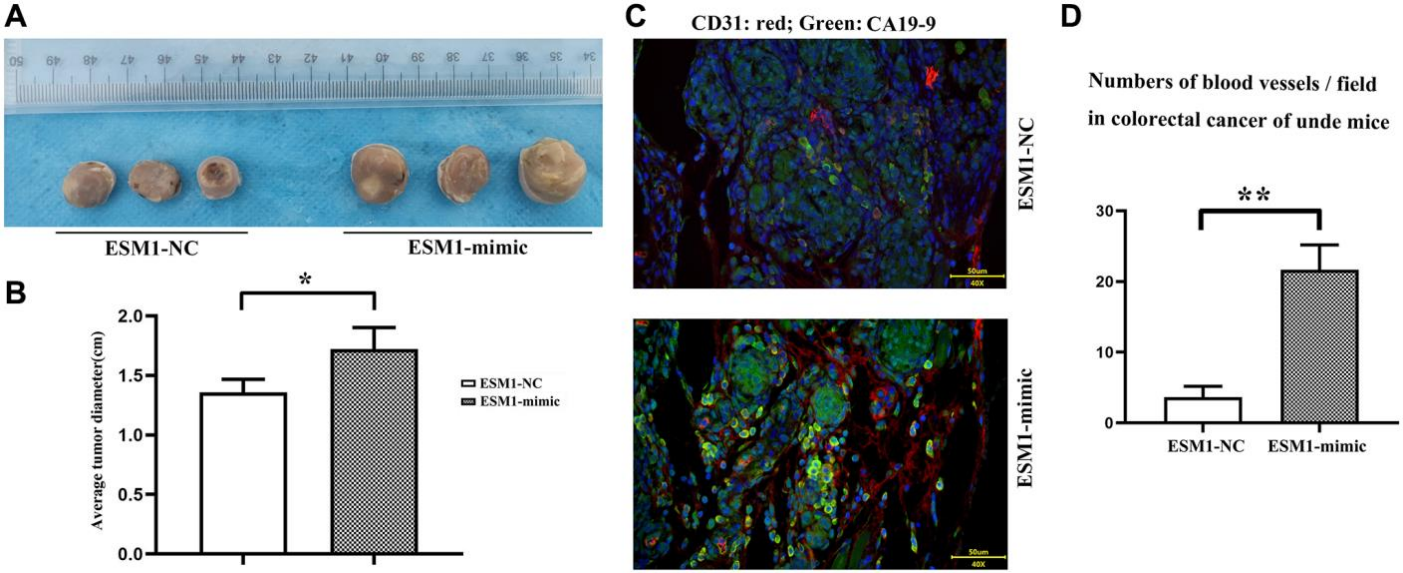
**ESM1 promotes the occurrence and development of tumors in nude mice.** (**A**) SW480 cells transfected with ESM1-NC and ESM1-mimic are subcutaneously injected into nude mice to generate tumors. (**B**) Statistics for tumor volume in nude mice. (**C**) Immunofluorescence double stain result plot.green:CA199, red:CD31; (**D**) Compared with the ESM1-NC group, CA199 and CD31 were significantly higher in the ESM1-mimic group. ^*^p < 0.05, ^**^p < 0.01.

The untreated SW480 cells were implanted with ESM1-mimic-treated SW480 cells into nude mice. Through the measurement of tumor diameter, it was found that ESM1 can promote the increase of tumor diameter (Figure 8A, 8B).

### ESM1 promotes the production of vascular and colorectal cancer cells

As shown in Figure 8C, CA199 expression is FITC-labeled green, CD31 expression is Alexa Fluor^®^ 647-labeled red, through observation, we can clearly find that compared to ESM1-NC, ESM1-mimic represents colorectal cancer cells CA199 and CD31 representing blood vessels are significantly elevated (Figure 8C, 8D).

### ESM1 might affect proliferation of CRC cells through the PI3K/Akt/mTOR pathway

**Figure 9:**
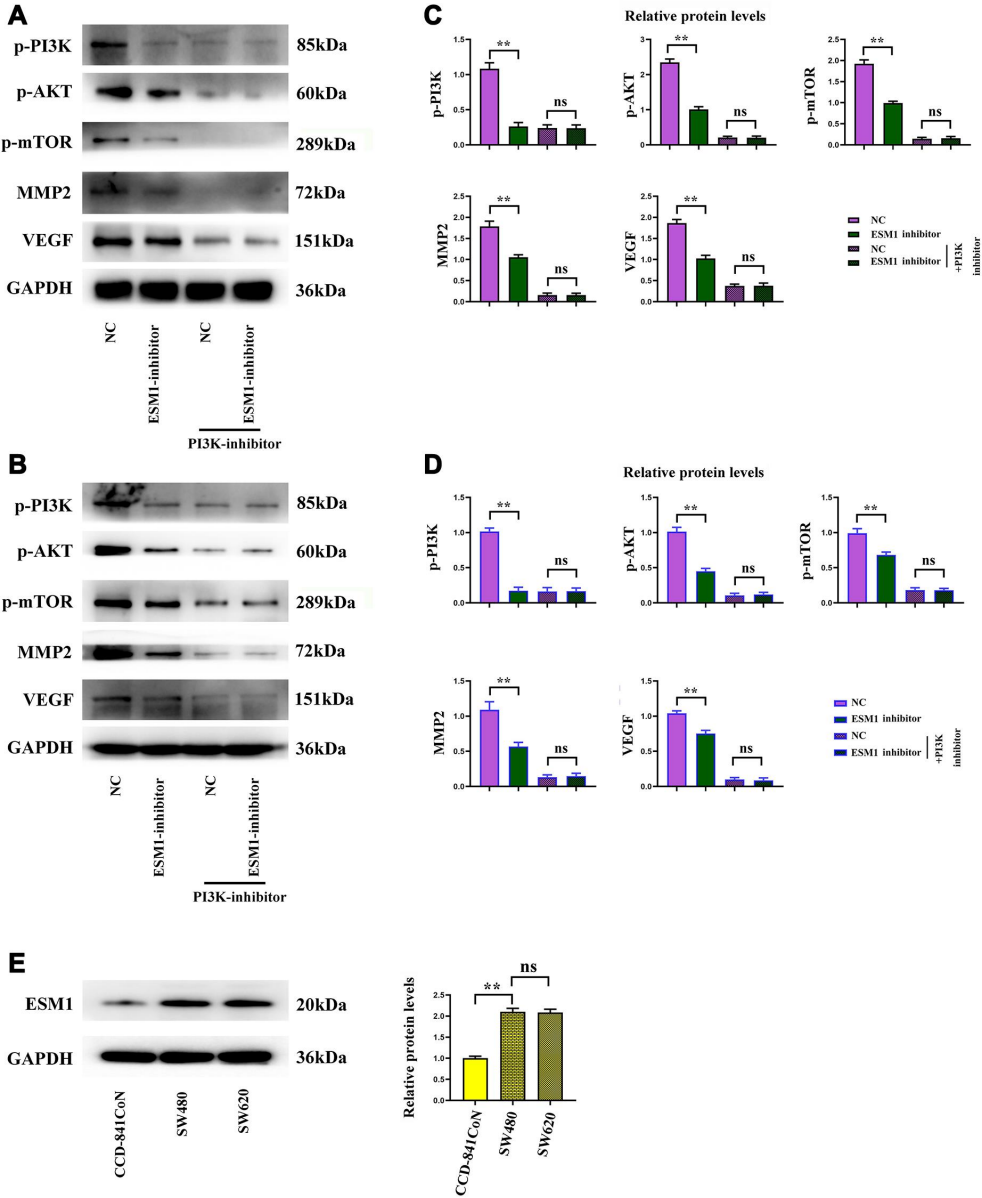
**ESM1 shows an elevated expression in CRC cells and promotes the proliferation of CRC cells.** (**A**, **B**) Relative protein expressions of p-PI3K, p-Akt, p-mTOR, MMP-2 and VEGF in ESM1-NC group, ESM1-mimic group, ESM1-NC + PI3K inhibitor group and ESM1-mimic + PI3K inhibitor group. (**C**, **D**) Expression of ESM1 in SW480 and SW620 cells. (**E**) Relative protein expression of ESM1 in CCD-841CoN, SW480 and SW620 cells. ^**^p < 0.01, ns: *p* > 0.05.

**Figure 10:**
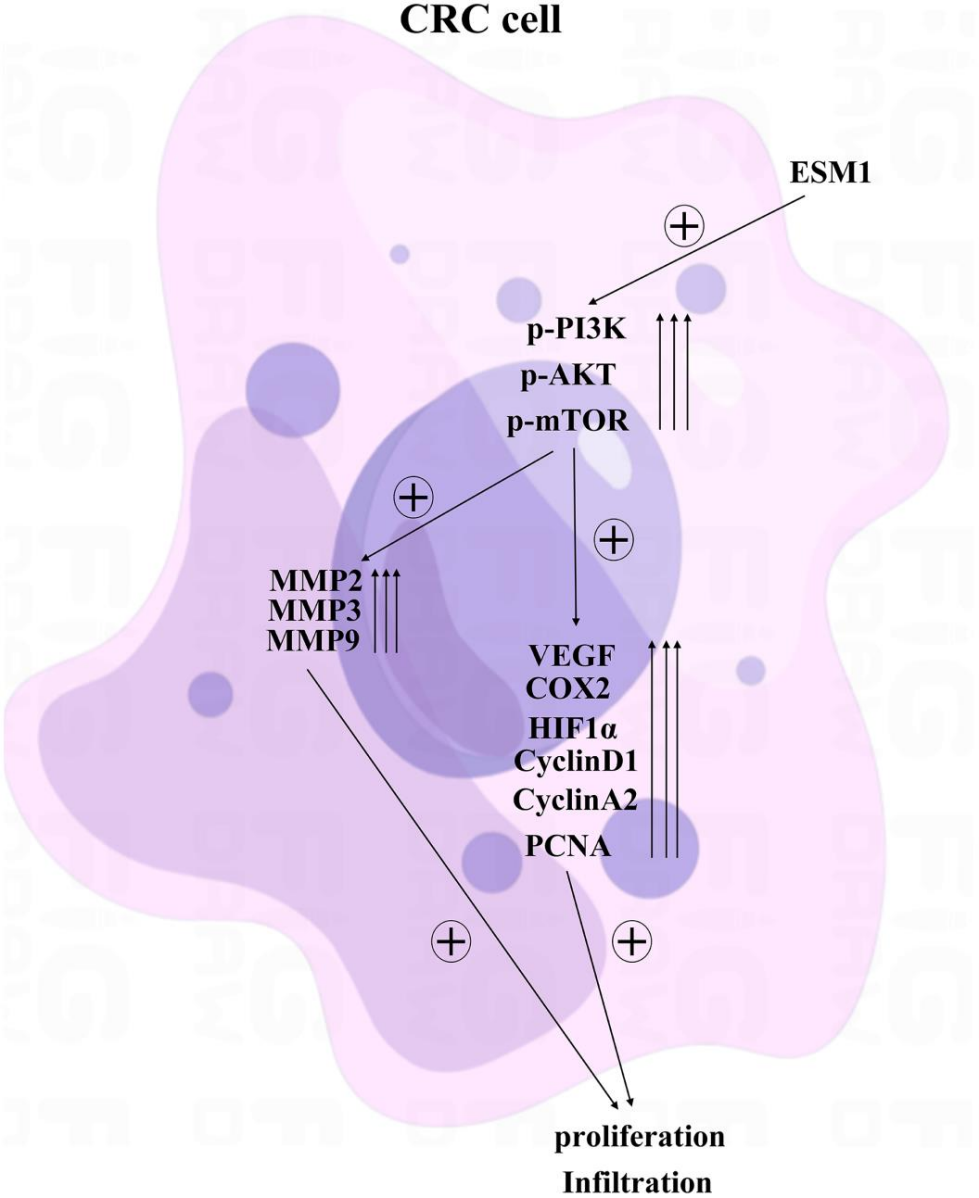
ESM1 may affect the proliferation of CRC cells through the PI3K/Akt/mTOR pathway.

The complementary Western blotting assay verified that the expression of ESM1 was indeed elevated in CRC cells. The protein expressions of p-PI3K, p-Akt, p-mTOR, MMP-2 and VEGF in ESM1-NC group were significantly higher than those in ESM1-inhibitor group, and the protein expressions in ESM1-NC group and ESM1-inhibitor group were inhibited after the addition of PI3K inhibitor (Figure 9A–9D). And the expression of ESM1 in SW480 and SW620 was significantly higher than that in CCD-841CoN (Figure 9E). This illustrates that ESM1 may affect the proliferation of CRC cells through the PI3K/Akt/mTOR pathway (Figure 10).

## DISCUSSION

CRC is mainly treated by surgery, but metastasis, recurrence and poor prognosis readily occur after surgery, so investigating its occurrence and development mechanism is of great significance for diagnosis, treatment and prognosis evaluation [22]. Based on the established analysis algorithm, the effective candidate biomarkers ESM1, CTHRC1 and AZGP1 have been identified from the blood, urine and saliva of patients with CRC, contributing to a better understanding of the molecular mechanism of CRC and providing a theoretical basis for clinical treatment [23]. Invasion and metastasis as major malignant biological features of tumor cells are considered to be predisposing factors for poor prognosis and serve as principal causes of failure in clinical anti-tumor treatment or even deaths [24]. Invasion and metastasis of malignancies involve complex processes regulated by multiple factors and genes, and suppressing invasion and migration of tumor cells is greatly conductive to improving patients’ quality of life [25]. It was revealed in this study that ESM1 promoted angiogenesis in CRC by activating the PI3K/Akt/mTOR pathway, thus accelerating tumor progression, consistent with previous studies. ESM1 is verified to be highly expressed in partial renal diseases, vascular inflammation, pneumonia and multiple tumors, and its crucial role in angiogenesis and tumor progression has also been confirmed.

The clinical research displayed that the expression level of ESM1 at the tumor site in patients with CRC is correlated with cancer differentiation [26]. In CRC cells, inhibiting ESM1 expression using siRNA is able to attenuate tumor cell invasiveness, and ESM1-siRNA can restrain tumor metastasis by modulating MMP proteins and EMT-related genes [27], consistent with findings reported in this study. After up-regulation of ESM1, the distance of SW480 and SW620 cell lines migrating to the scratch center rose notably, and the number of migrating cells, basement membrane-penetrating cells and colonies formed was increased overtly. Consistently, Western blotting revealed that in contrast to those in ESM1-NC group, the protein expressions of MMP-2, MMP-3, MMP-9, Cyclin D1 and Cyclin A2 in SW480 and SW620 cells of ESM1-mimic group were overtly elevated. MMPs are reported to act as essential regulators in the migration, growth and inflammation of cancer cells. MMP-9 is implicated in the inositol hexaphosphate-induced migration and invasion of CRC cells [28]. MMP-9 as an important proteolytic enzyme is expressed multiple tumors and enhances cell invasion and metastasis abilities by degrading extracellular matrix, thus facilitating tumor cell invasion and migration [29, 30]. Besides, studies have confirmed that Cyclin A2 is highly expressed in multiple tumor tissues, but not or lowly expressed in normal tissues [31, 32]. Cyclin D1 is viewed as an important cell cycle gene mainly acting on the G1 phase and is capable of modulating cell cycle. Its high expression promotes cell proliferation and differentiation, which is closely associated with the occurrence and development of tumors [33]. For these reasons, it is concluded that ESM1 can enhance the migration, invasion and proliferation abilities of SW480 and SW620 cell lines. Numerous studies have shown that COX-2 promotes angiogenesis through a variety of mechanisms. For example, increased COX-2 expression is strongly associated with enhanced VEGF mRNA or protein production. VEGF is one of the most potent angiogenic factors, which can promote endothelial cell proliferation and migration and induce vascular permeability. The induction of VEGF is an important component of vascular reactivity and angiogenesis, and HIF-1α is known to be a key transcription factor in this process. A growing body of research has confirmed that COX-2 is upregulated along with HIF-1α and VEGF during hypoxia.

Furthermore, it was also uncovered that ESM1 could facilitate angiogenesis in CRC. The results of tube formation assay indicated that the supernatant of SW480 and SW620 cells with ESM1 overexpression could promote angiogenesis in 3B-11 cells. In comparison with those in ESM1-NC group, the expressions of angiogenesis-related proteins VEGF, COX-2 and HIF-1α were prominently raised in SW480 and SW620 cells in ESM1-mimic group. Tumor neovascularization has been proven to provide tumor cell growth with nutrients and serves as a crucial path of tumor metastasis. Hence, ESM1 that promotes the angiogenesis in CRC will further accelerate the occurrence and development of tumors.

Biological behavior experiments on SW480 and SW620 cells demonstrated that ESM1 could promote angiogenesis in CRC and accelerate the migration, invasion and proliferation of CRC cells. Combined with results of bioinformatics analysis, the mechanism of action was further investigated via intervention with PI3K inhibitor. The results revealed that the protein expressions of p-PI3K, p-Akt and p-mTOR were decreased evidently, and the protein expressions of MMP-2, MMP-3, MMP-9, Cyclin D1, Cyclin A2, VEGF, COX-2 and HIF-1α subsequently declined. In conclusion, ESM1 may promote angiogenesis in CRC by activating the PI3K/Akt/mTOR pathway, thus accelerating tumor progression.

## Supplementary Materials

Supplementary Figure 1
