# Supplementary material for: ESM1 promotes angiogenesis in colorectal cancer by activating PI3K/Akt/mTOR pathway, thus accelerating tumor progression
Source: Aging (Albany NY). 2023 Mar 7;15(8):2920–36. doi: 10.18632/aging.204559 (PMC10188330; doi:10.18632/aging.204559)
Supplement: Supplementary Figure 1 [file aging-15-204559-s001.pdf]

SUPPLEMENTARY FIGURE

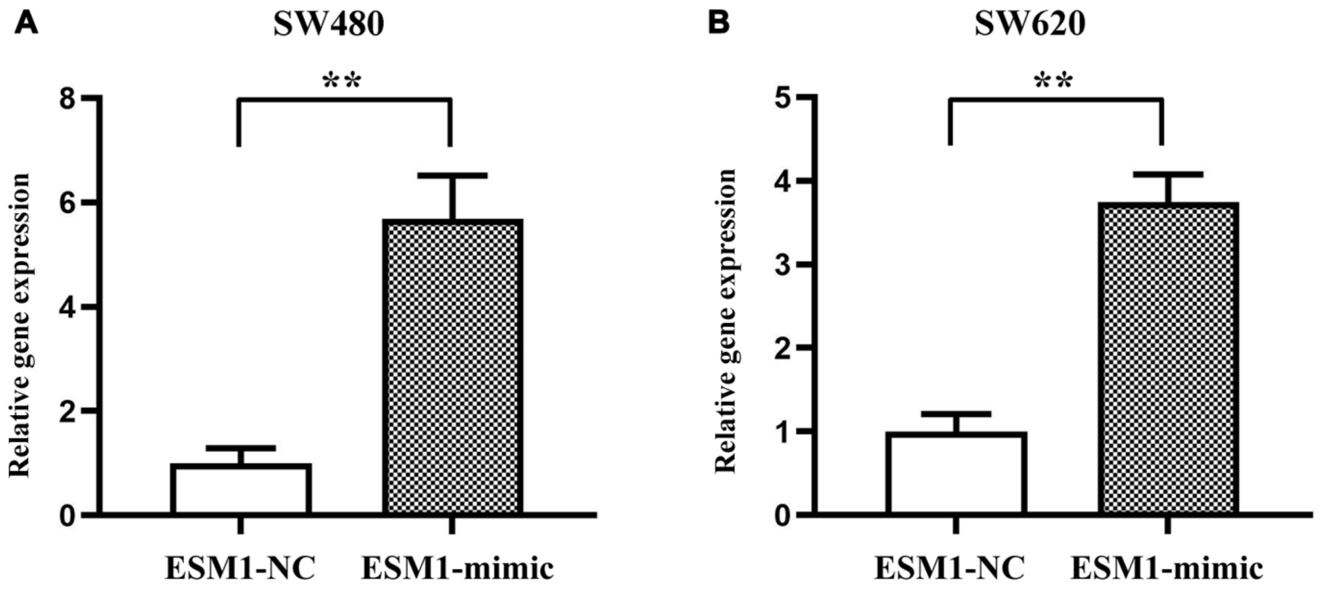

Supplementary Figure 1. Q-PCR verified that ESM1 transfection was successful. (**A**, **B**) Expression of ESM1 in the ESM1-NC and ESM1-mimic groups in sw480 and sw620 cells. The expression of ESM1 in the ESM1-mimic group increased significantly relative to the ESM1-NC group. $^{**}P < 0.01$.
